# Electroacupuncture Regularizes Gastric Contraction and Reduces Apoptosis of Interstitial Cells of Cajal in Diabetic Rats

**DOI:** 10.3389/fphys.2021.560738

**Published:** 2021-04-01

**Authors:** Hongcai Wang, Kaile Zhao, Ning Shi, Qiong Niu, Chengxia Liu, Yan Chen

**Affiliations:** ^1^Department of Neurology, Binzhou Medical University Hospital, Binzhou, China; ^2^Department of Gastroenterology, Binzhou Medical University Hospital, Binzhou, China

**Keywords:** electroacupuncture, apoptosis, interstitial cells of cajal, IGF-1/IGF-1R pathway, Nrf2/HO-1 pathway

## Abstract

**Background/Aims:**

Gastric dysmotility is a frequent complication among patients with diabetes mellitus. Electroacupuncture (EA) has been empirically used to relieve gastrointestinal symptoms. The aims of this study were to investigate the effects of EA on gastric contraction and the mechanisms of interstitial cells of Cajal (ICC) involved.

**Materials and Methods:**

Male Sprague–Dawley rats were randomized into the normal control, diabetes (DM), diabetic and sham EA (DM + SEA), diabetic and low-frequency EA (DM + LEA), and diabetic and high-frequency EA (DM + HEA) groups. Diabetic models were established and then treated with EA for 8 weeks. Body weight and blood glucose were recorded every 2 weeks. The spontaneous contractions of distal gastric strips were analyzed. Immunostaining and RT-PCR were used to test the apoptotic ICC, IGF-1/IGF-1R, and Nrf2/HO-1 pathways.

**Results:**

The body weight in the DM + LEA and DM + HEA groups were increased compared with that of the DM group, though there was no effect on the blood glucose. The gastric contractions were obviously disordered in the DM group, but EA could regularize the contractions. The number of apoptotic ICC was dramatically increased in the DM group, but reduced with EA treatment. Meanwhile, the IGF-1/IGF-1R pathway was verified to be significantly altered in diabetic rats. The Nrf2/HO-1 pathway was not significantly increased in the DM group. EA with different frequencies efficiently improved the expression of IGF-1/IGF-1R signaling and activated the Nrf2/HO-1 pathway.

**Conclusion:**

EA could improve gastric motility dysfunction and attenuate ICC apoptosis possibly through the regulation of IGF-1/IGF-1R and Nrf2/HO-1 pathways. EA may be a potential therapeutic method for diabetic gastric motility dysfunction.

## Introduction

Acupuncture is a common alternative medicine empirically practiced for more than 2,500 years to treat different diseases (van den Berg-Wolf and Burgoon, 2017). Electroacupuncture (EA) is a modified application of acupuncture, which is more popular for its parameter quantization. As a form of traditional Chinese medicine, the efficacy of EA in pain relief was accepted by the United States and other Western countries (Ju et al., 2017). The mechanisms of EA to relieve pain are explained by releasing endogenous opioid peptides in the brain and spinal cord to mediate the analgesic effect of EA (Wan et al., 2018). According to traditional Chinese medicine, *Zusanli acupoint* (ST36) is commonly used to treat gastrointestinal disorders, such as motor dysfunction, visceral pain, and secretion (Chen et al., 2018; Song et al., 2019; Guo et al., 2020). ST36 is located laterally below the knee about 5 mm beside the anterior tubercle of the tibia (Yang et al., 2020). In the past years, numerous studies have manifested that EA at ST36 has been widely accepted to treat diabetic patients with symptoms such as vomiting, nausea, abdominal bloating, and abdominal pain (Wang, 2004; Liu et al., 2012; Pan et al., 2017). Unfortunately, the underlying mechanisms of EA at ST36 on diabetic gastric motility dysfunction have not yet been elucidated.

Up to now, an important mechanism of gastroparesis is thought to be the defect and loss of interstitial cells of Cajal (ICC), which could produce slow wave potential and mediate neurotransmission between enteric nerve system and smooth muscles (Grover et al., 2019). Ordög et al. (2000) found that the diabetic mice developed obviously slowed gastric emptying, damaged electrical activity, and abnormal neural transmission accompanied with damage to ICC. Similarly, ICC are absent in patients with gastroparesis up to a third, and loss of ICC leads to aberrant gastric slow waves and exacerbates the original gastrointestinal symptoms (Forster et al., 2005). Our previous study (Chen et al., 2013a) also showed that apoptotic ICC were observed in the distal stomach of diabetic rats, and EA at ST36 could renovate the impaired networks of ICC though preventing the apoptosis. However, the possible mechanism of EA at ST36 participating in the apoptosis of ICC is still unclear.

Insulin-like growth factor 1 (IGF-1) is well-known as a hormone playing growth-promoting effects on the majority of cells, and binding to its specific receptor (IGF-1R) initiates intracellular signaling (Laviola et al., 2007). Horváth et al. reported that insulin and IGF-I receptors were not expressed in ICC, but reduced insulin/IGF-I signaling resulted in deficiency of ICC in diabetes (Horváth et al., 2005, 2006). Otherwise, it is reported that EA was available to alleviate cerebral ischemic damages through improving the expression of IGF-1 in monkeys (Gao et al., 2006). At the same time, it is also documented that EA facilitated IGF-1 expression in the ovariectomized osteoporosis rats (Feng et al., 2008). Our previous work manifested that long-pulse gastric electrical stimulation could restore IGF-1 signaling to protect ICC in diabetic gastroparesis models (Li et al., 2016). However, the relevant effects of EA on IGF-1/IGF-1R signaling in diabetic rats need to be further investigated.

Heme oxygenase-1 (HO-1), a widely existing antioxidant defense enzyme, protected against cell death by the suppression of apoptosis, inflammation, and cell proliferation in various models (Waza et al., 2018). Nuclear factor erythroid 2-related factor (Nrf2), combined with HO-1, is a multifunctional regulator for anti-oxidant and anti-inflammatory effects (Loboda et al., 2016). Previous studies showed that the expression of HO-1 was downregulated resulting in the destruction of the ICC network and delayed gastric emptying in diabetic models; furthermore, increased HO-1 improved the injury of ICCs and delayed gastric emptying (Choi et al., 2008; Mogami et al., 2013). Simultaneously, it was found that EA could improve the expression of HO-1 and ICC in the stomach of diabetic mice (Tian et al., 2018). Nevertheless, the effects of EA on the Nrf2/HO-1 pathway need further discussion.

The aims of the present study were to explore the effects and mechanisms of EA on the gastric contraction and the apoptosis of ICC, specifically to illuminate IGF-1/IGF-1R and Nrf2/HO-1 pathways whether they were involved in the maintenance of ICC.

## Materials and Methods

### Animals

Ethical approval from the Binzhou Medical University Hospital Animal Ethics Committee was obtained for using rats. All experimental procedures were conducted according to the ethical guidelines from the Animal Care and Use Committee and Laboratory Animal Ethical Committee of Binzhou Medical University Hospital. Eight-week-old male Sprague–Dawley rats (*N* = 50) were bought from Jinan Pengyue Experimental Animal Breeding Co., Ltd. (Shandong, China) and then were housed in the standard laboratory conditions with a constant temperature (22 ± 0.5°C) and 12-h light/dark cycle. After adaptive feeding for 1 week, the rats were randomly grouped into the normal control, diabetes (DM), diabetic and sham EA (DM + SEA), diabetic and low-frequency EA (DM + LEA), and diabetic and high-frequency EA (DM + HEA) groups.

### Diabetic Model

Diabetic models were developed by i.p. injection of streptozotocin (STZ, 60 mg/kg, Sigma, St. Louis, MO, United States) dissolved in citrate buffer solution (pH 4.5; Sigma, St. Louis, MO, United States), which is closer to Type 1 diabetes (Chen et al., 2013a). The normal control group was given an equal volume of the vehicle. After the STZ or vehicle injection, all of the rats were deprived of water for 4 h. After a week, blood glucose in the tail vein was tested with a glucometer, and induction of diabetes was determined twice with a random blood glucose ≥ 16.7 mmol/L. Body weight and blood glucose were measured every 2 weeks.

### Experimental Protocol

Rats in the DM + SEA, DM + LEA, and DM + HEA groups were given EA stimulation with needles inserting into the bilateral ST36 acupoints 30 min per day for 8 weeks. A G6805-2A stimulator (Shanghai Huayi Medical Instrument Factory, Shanghai, China) was used in this experiment. The parameters of EA were chosen as 10 Hz, 1–3 mA for low frequency EA and 100 Hz, 1–3 mA for high frequency EA according to the previous study (Chen et al., 2013b). The final electric current was determined on the basis of slight tremor of the lower limbs. The sham EA stimulation was performed by only acupuncture without electrical current. After EA for 8 weeks, the rats were euthanized to collect the strips of gastric antrum for the mechanical contractility study, and tissues for RT-PCR and immunostaining.

### Spontaneous Contraction of Gastric Circular Muscle Strips

After the rat was sacrificed, the entire stomach was taken out immediately and opened along the greater curvature. Circular muscle strips about 7 × 2 mm were obtained from the gastric antrum with the mucosal layer removed and then immersed in Krebs solution bubbled with 95% O_2_/5% CO_2_. The strips were suspended in an organ bath full of Krebs buffer kept at 37°C and persistently inflated with 95% O_2_/5% CO_2_. The strip’s tension was recorded using isometric force transducers (Fort-10, WPI, Sarawsota, FL, United States) connected to a recorder (MP-100 system). Data were digitized and analyzed with software named Acknowledge 3.7.1 (Biopac Systems, Santa Barbara, CA, United States). Primarily, a tension of 1 g was chosen to adjust the contraction, and a period about 60 min was prepared for the strips to equilibrate following formal recording of the spontaneous contraction of gastric circular muscle strips.

### ELISA for Insulin-Like Growth Factor 1

Blood samples were obtained from the abdominal aortic vein and then processed through a 15-min centrifugation at 3,000 rpm. The plasma was kept at the freezer as soon as possible. The protein of IGF-1 concentration was tested by an ELISA kit (RayBiotech, United States) according to the manufacturer’s protocols. Optical density (OD) of the samples and the standard was measured on an ELISA plate scanner at 450 nm.

### Immunostaining

After fixing in 4% paraformaldehyde, the tissue was made into paraffin blocks and cut into slices of 4–6 μm. The slices were put into xylene to deparaffinize two times and successively hydrated in a series of graded alcohols. To block the endogenous peroxidase activity though 0.3% hydrogen peroxide, the sections were kept in a dark box to avoid light. After antigens were exposed by microwave and non-specific reaction by normal rabbit serum was blocked, the primary antibody including c-kit (1:100, Santa Cruz, United States), IGF-1R (1:100, Abcam, United States), HO-1 (1:100, Abcam, United States), and Nrf2 (1:100, Abcam, United States) were incubated and kept at 4°C overnight.

For immunohistochemistry staining of IGF-1R, HO-1, and Nrf2, horseradish peroxidase- (HRP)-linked streptavidin-marked secondary antibody was incubated for 1 h. To show the positive protein, a fresh 3,3°C. TUNEL Kit (enzyme solution: labeling solution = 1:9, Roche, Germany) was applied to detect apoptotic cells. DAPI was incubated for 5 min to stain the nucleus. Anti-fluorescence quenching agent was added to protect the fluorescence and slices were observed using a fluorescence microscope. Image J software was employed to calculate the immunofluorescence staining of c-kit + /TUNEL + cells.

### Real-Time Quantitative Reverse-Transcription PCR

Total RNA of gastric antrum was extracted using the TRIzol reagent and reverse-transcribed into cDNA with Prime Script RT Master Mix (Takara, Otsu, Japan) according to the manufacturer’s instructions. The primers we used are shown in Table 1. The reaction system was 20 μl in volume, including 10 μl of SYBR green, 1 μl of forward primer and 1 μl of reverse primer, and 2 μl of diluted cDNA and 6 μl of ddH_2_O. The next step was a heating cycle of 95°C for 10 min, and then a circulation was processed 40 times at 95°C for 15 s and 60°C for 1 min. A melt curve was gained by ascending the temperature. At the end, cycle threshold (CT) values were obtained according to fluorescent signals. The gene transcript level was controlled to the housekeeping gene GAPDH. The –2ΔΔCT method was used to determine the relative expression of the desired gene.

**TABLE 1:** Primer sequences designed for RT-PCR.

Gene	Primer	5′ → 3′	Size (bp)
IGF–1R	Forward	5′-CTACCTCCCTCTCTGGGAATG-3′	150
	Reverse	5′-GCCCAACCTGCTGTTATTTCT-3′	
HO-1	Forward	5′-GCATGTCCCAGGATTTGTCC-3′	192
	Reverse	5′-GGTTCTGCTTGTTTCGCTCT-3′	
Nrf2	Forward	5′-CCCATTGAGGGCTGTGAT-3′	247
	Reverse	5′-TTGGCTGTGCTTTAGGTC-3′	
GAPDH	Forward	5′-ACAGCAACAGGGTGGTGGAC-3′	253
	Reverse	5′ -TTTGAGGGTGCAGCGAACTT-3′	

*IGF-1R, insulin-like growth factor-1 binding receptor; HO-1, heme oxygenase-1; Nrf2, nuclear factor erythroid 2-related factor; GAPDH, housekeeping gene.*

### Statistical Analysis

All data were presented as mean ± standard error of the mean (SEM), and analysis of variance (ANOVA) was applied to compare statistical differences among multiple groups. Two-way ANOVA was used to analyze the data of the animals’ body weight and blood glucose, and one-way ANOVA to the rest of the data. *Post hoc* tests were used after a one-way or two-way ANOVA to compare all groups with each other. The LSD *post hoc* test was conducted when equal variances were assumed, and the Dunnett T3 *post hoc* test was applied when unequal variances were assumed. A P value less than 0.05 was adopted as a statistically significant difference. Statistical analyses were processed by SPSS version 24.0 (SPSS Inc., Chicago, IL, United States).

## Results

### Body Weight and Blood Glucose

**FIGURE 1:**
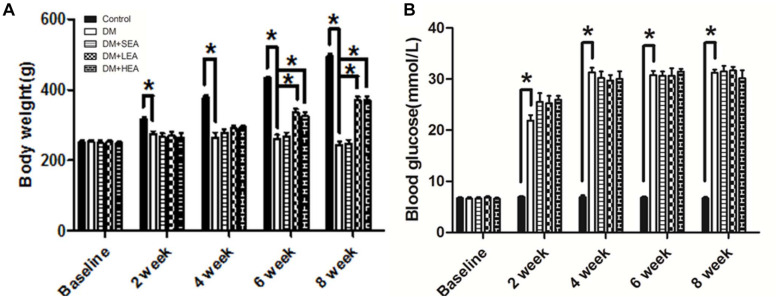
Body weight and blood glucose level were measured in the whole experiment. **(A)** Compared with the diabetes (DM) group, the body weight of the DM + low-frequency electroacupuncture (LEA) and DM + high-frequency electroacupuncture (HEA) groups was increased and gained markedly at the end of 6, 8 weeks (all *P* ≤ 0.001). **(B)** The blood glucose levels of the diabetic rats were significantly increased from 2 to 8 weeks. Additionally, no significant differences were found between the DM group and the DM + EA group at all time points (all *P* > 0.05). Two-way ANOVA was used to analyze the data of the body weight and blood glucose. The LSD *post hoc* test was used for the analyses. *P < 0.05 compared with the DM group.

Although there was no significant difference at the baseline among the groups (Figure 1A), the body weight in the DM group was markedly decreased at the end of 2 and 4 weeks compared with that in the control group (2 weeks: 274.250 ± 7.282 vs. 316.250 ± 5.467, *P* = 0.003; 4 weeks: 278.625 ± 9.852 vs. 377.250 ± 6.094, *P* < 0.001). However, no significant differences were observed between the diabetic rats and those treated with EA (all *P* > 0.05). At the end of 6 and 8 weeks, the body weight of the DM group was obviously reduced compared with the normal rats (all *P* < 0.001), but the body weight in the DM + LEA and DM + HEA groups were all significantly increased (compared with the DM group, all P ≤ 0.001).

The blood glucose levels of these rats from the five groups were similar at the baseline. However, the blood glucose levels of diabetic rats were significantly increased in the following time. No significant differences were found afterward between the DM group and the DM + EA group (Figure 1B).

### Effects of Electroacupuncture at Zusanli Acupoint on Spontaneous Contraction of Gastric Circular Muscle Strips

**FIGURE 2:**
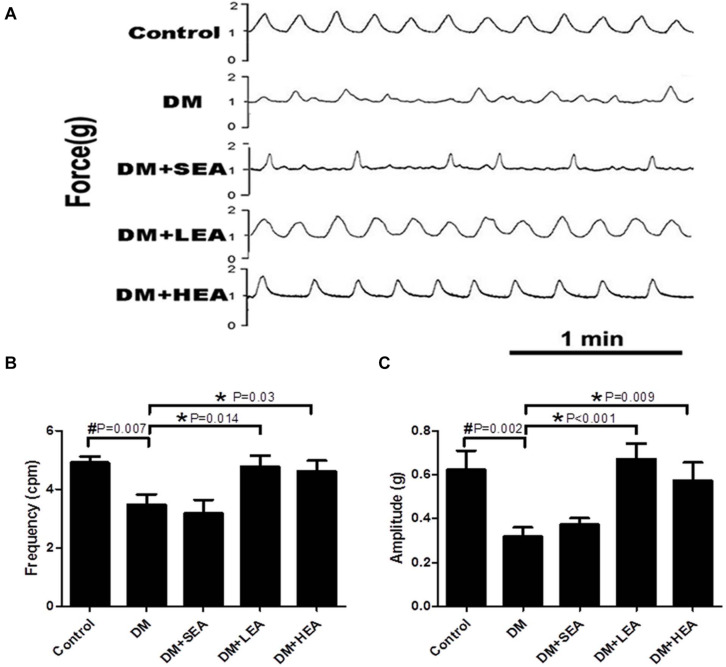
Spontaneous contraction of gastric circular muscle strips in different groups. **(A)** The representative traces of spontaneous contraction wave of the gastric antrum smooth muscle strips in each group. **(B)** The frequency of spontaneous contraction wave in each group, and **(C)** reveals the amplitude of spontaneous contraction wave in each group. One-way ANOVA was used to analyze the data, and the Dunnett T3 *post hoc* test was used for the analyses of the body weight (4 and 6 weeks), with the LSD *post hoc* test for the analyses of the body weight (0, 2, and 8 weeks) and the blood glucose. *P < 0.05 compared with the DM group, ^#^*P* < 0.05 compared with the control group.

As shown in Figure 2, the amplitude and frequency of spontaneous contraction in the DM group were damaged, and rhythm disorders were performed (*P* = 0.002 and *P* = 0.007 vs. the control group). In the DM + SEA group, the spontaneous contractions were still disordered with abnormal frequency and amplitude (*P* = 0.563 and *P* = 0.575 vs. the DM group). Both LEA and HEA stimulation significantly improved the amplitude and frequency of spontaneous contraction of muscle strips compared with those in the diabetic rats (LEA: *P* < 0.001 and *P* = 0.014; HEA: *P* = 0.009 and *P* = 0.030).

### Effects of Electroacupuncture at Zusanli Acupoint on Apoptotic Interstitial Cells of Cajal

**FIGURE 3:**
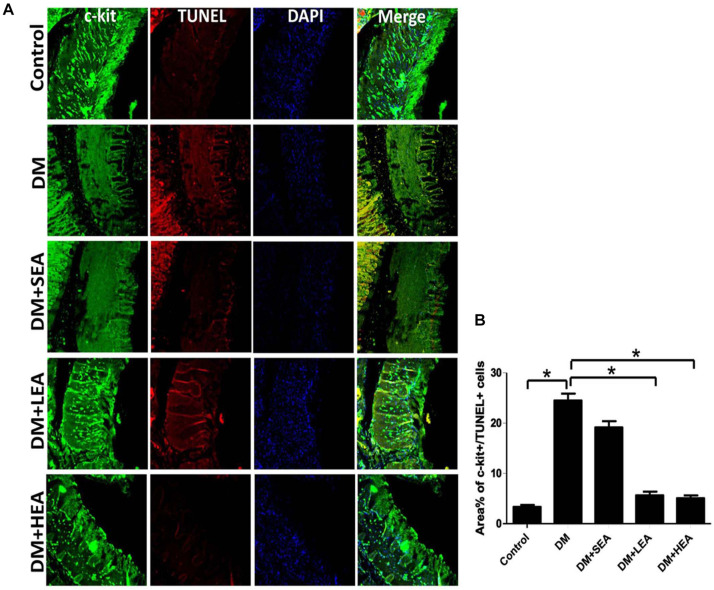
Confocal micrographs of apoptotic interstitial cells of Cajal (ICC) labeled with c-kit (green), TUNEL (red), and DAPI (blue). **(A)** In the control group, many c-kit + cells were observed in the gastric wall, but a few of c-kit + /TUNEL + cells were found. The number of ICC was decreased in the DM and diabetic and sham EA (DM + SEA) group. A few apoptotic ICC was seen in the DM + LEA and DM + HEA groups. Scale bars = 20 mm. **(B)** Quantification of c-kit + /TUNEL + cells in the stomach of each group. One-way ANOVA was used to analyze the data, and the Dunnett T3 *post hoc* test was used for the analyses. *P < 0.05 compared with the DM group.

The apoptotic ICC in different groups are displayed in Figure 3. Numerous c-kit + cells with a small quantity of TUNEL + cells were found in the intramuscular and muscular layer of the antrum in the control group. However, many TUNEL + cells with reduced c-kit-positive cells were shown both in the DM and DM + SEA groups (both *P* < 0.001 vs. the control group). There were plenty of ICCs with rare TUNEL + cells recognized in the DM + LEA and DM + HEA groups (both *P* < 0.001 vs. the DM group).

### Effects of Electroacupuncture at Zusanli Acupoint on the Plasma Insulin-Like Growth Factor 1 Level

**FIGURE 4:**
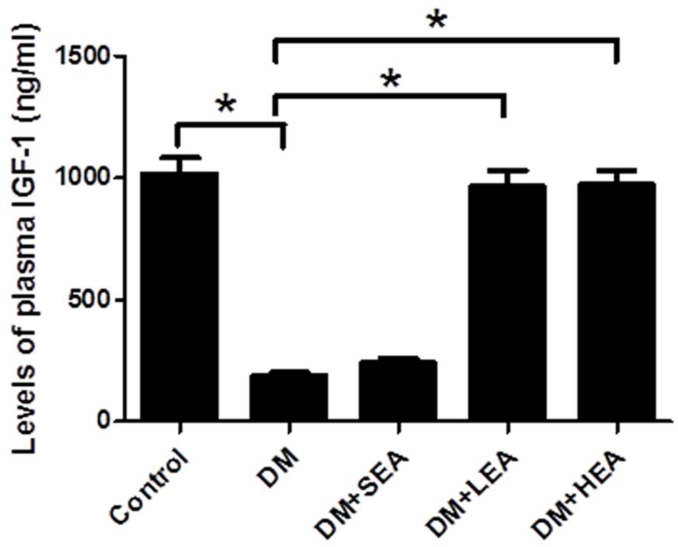
The level of plasma insulin-like growth factor-1 (IGF-1) in each group. The IGF-1 level in the DM group was lower than that in the control group. However, the IGF-1 level in the DM + LEA and DM + HEA groups was elevated. One-way ANOVA was used to analyze the data, and the LSD *post hoc* test was used for the analyses. *P < 0.05 compared with the DM group.

As indicated in Figure 4, the plasma IGF-1 level was obviously decreased in the DM group compared with that in the control group (190.257 ± 13.305 vs. 1,018.483 ± 65.996, *P* < 0.001). No significant difference was found between the DM group and the DM + SEA groups (*P* = 0.464). Otherwise, the plasma IGF-1 level was dramatically elevated by LEA and HEA (both *P* < 0.001 vs. the DM group).

### Effects of Electroacupuncture at Zusanli Acupoint on the Expression of IGF-1R

**FIGURE 5:**
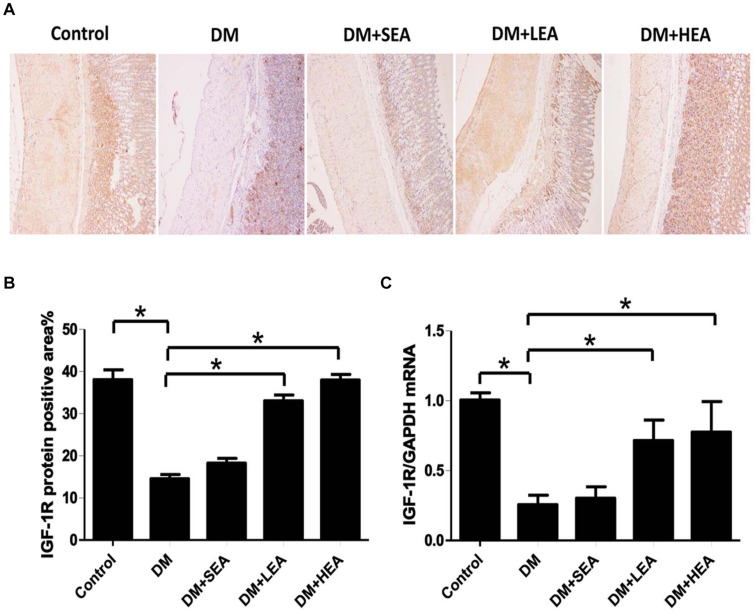
The expression of IGF-1R in each group. **(A)** Immunohistochemical technique showed that lots of IGF-1R + cells in the control group, but in the DM and DM + SEA groups, there were few IGF-1R + cells distributed in the intramuscular layer (100 ×). The expression of IGF-1R + cells in the DM + LEA and DM + HEA groups was improved. **(B)** Quantification of IGF-1R expression in the stomach of each group. **(C)** RT-PCR revealed that the IGF-1R mRNA expression in the DM group was significantly decreased compared with that in the normal control group. In the DM + LEA and DM + HEA groups, expression of IGF-1R mRNA was increased compared with that in the DM group. One-way ANOVA was used to analyze the data of expression of IGF-1R protein and mRNA, and the LSD *post hoc* test was used for the analyses. *P < 0.05 compared with the DM group.

The expression of IGF-1R in each group was reflected by the immunohistochemistry and RT-PCR in Figure 5. Figures 5A,B indicated a lot of IGF-1R + cells locating in the muscular and intramuscular layer of the control group, but in both the DM and DM + SEA groups, there were few IGF-1R + cells present in the intramuscular layer (both *P* < 0.001 vs. the control group). However, the expressions of IGF-1R + cells were distinctly improved both in the DM + LEA and DM + HEA groups (both *P* < 0.001 vs. the DM group).

Similarly, the mRNA expression of IGF-1R in the DM group was dramatically decreased in Figure 5C (*P* = 0.002 vs. the control group). The expressions of IGF-1R mRNA in the DM + LEA and DM + HEA groups were distinctly increased (*P* = 0.031 and *P* = 0.017 vs. the DM group).

### Effects of Electroacupuncture at Zusanli Acupoint on the Expression of Nuclear Factor Erythroid 2-Related Factor/Heme Oxygenase-1 Pathway

**FIGURE 6:**
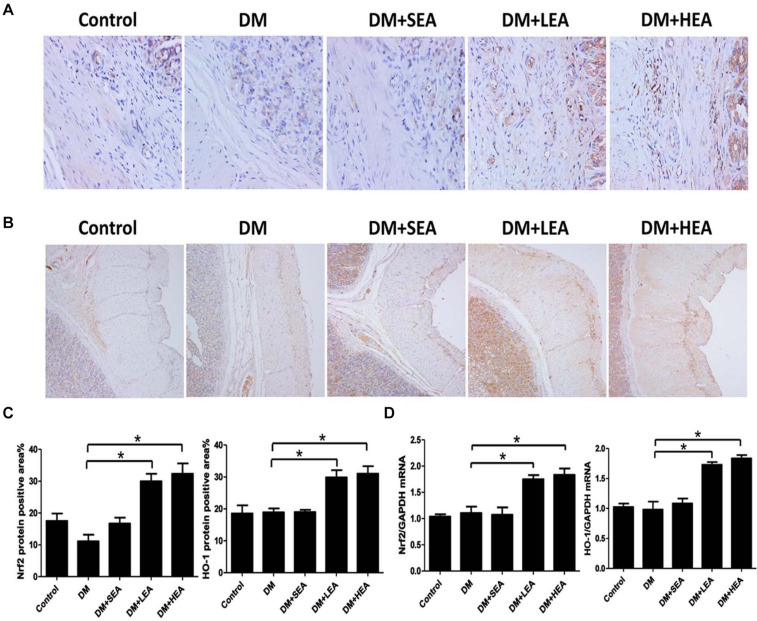
The effects of electroacupuncture (EA) on the expression of nuclear factor erythroid 2-related factor/heme oxygenase-1 (Nrf2/HO-1) pathway. **(A)** The expression of Nrf2 was increased in the DM group compared with the normal control group, but it was significantly increased in the DM + LEA and DM + HEA groups (400×). **(B)** HO-1 in the DM group was elevated compared with the normal control group. In the DM + LEA and DM + HEA groups, the expression of HO-1 was increased compared with that in the DM group (100×). **(C)** Quantification of Nrf2 and HO-1 protein expressions in the stomach of each group. **(D)** The mRNA expressions of Nrf2 and HO-1 in the DM group were elevated compared with those in the normal control group. Compared with the DM group, the Nrf2 and HO-1 mRNA expressions were markedly increased in the DM + LEA and DM + HEA groups. One-way ANOVA was used to analyze the data, and the LSD *post hoc* test was used for the analyses. *P < 0.05 compared with the DM group.

Both Nrf2 and HO-1 expressions in the DM group were not elevated in Figure 6 (both *P* > 0.068 vs. the control group). Likewise, the expressions of Nrf2 and HO-1 in the DM + SEA group were not dramatically added in contrast to the DM group (*P* = 0.110 and *P* = 1.000). However, lots of Nrf2 + or HO-1 + cells could be seen in the diabetic rats with LEA and HEA groups (Nrf2: both *P* < 0.001; HO-1: *P* = 0.012 and *P* = 0.008).

The Nrf2 and HO-1 mRNA expressions in the DM group were elevated (*P* < 0.001 and *P* = 0.008 vs. the control group). No significant difference in the mRNA expressions of Nrf2 and HO-1 were found between the DM + SEA group and the DM group (*P* = 0.350 and *P* = 0.437). The Nrf2 and HO-1 mRNA expressions were obviously increased both in the DM + LEA and DM + HEA groups (all *P* < 0.001 vs. the DM group).

## Discussion

In this study, apoptosis of ICC was observed along with the decreased IGF-1/IGF-1R and Nrf2/HO-1 pathways in the gastric antrum of diabetic rats, resulting in disordered spontaneous contraction of gastric antrum. Further, we demonstrated that EA with different frequencies could reduce the apoptosis of ICC with improved IGF-1/IGF-1R pathway and increased Nrf2/HO-1 pathway, accompanying rhythmic contraction of the gastric antrum. These results revealed that EA could protect ICC from apoptosis to alleviate the contraction of gastric antrum, partly through the IGF-1/IGF-1R and Nrf2/HO-1 pathway.

Gastroparesis, namely, delayed gastric emptying, is a frequent complication of diabetic patients with gastrointestinal disorders, which was observed in our previous study (Ordög et al., 2000). However, regular phasic contraction of the antrum is an important factor to determine the gastric motility. Marques et al. (2014) showed that severe diabetes decreased mechanical amplitude and induced gastric dysrhythmia, but mean frequency of gastric contractions remained unchanged in type 1 diabetic rats. However, in our study, both the frequency and amplitude of spontaneous contraction were reduced. These can be explained by the recording methods that are different, and the contraction waves in the DM group are very irregularly shaped and unequal sized, so that some small irregular waves cannot be statistical. Therefore, disordered antrum contractions could be identified in diabetic rats with gastroparesis.

It is well known that ICC could play a key role in generation and propagation of slow waves and intermediate between nerves and smooth muscle cells to control motility. The peristaltic motor patterns showed distinct uncoordinated motor activities when there is a lack or deficiency of ICC (Wang et al., 2005). In our previous study, the mechanical contraction of gastric antrum induced by acetylcholine was severely affected with ICC loss in diabetic gastroparesis (Chen et al., 2013b). In this study, we focused on the spontaneous contraction of gastric antrum, which could manifest the physiological state of gastric contraction, and found that the frequency and amplitude of spontaneous contraction wave in diabetic rats were significantly decreased, but EA could improve the gastric contraction, reflecting that EA plays a role in alleviating gastric dysmotility.

It was an ongoing process of ICC apoptosis in the normal colon tissue of healthy humans, and the apoptotic ICC could be identified by immunolabeling for activated caspase-3, terminal deoxynucleotidyl transferase dUTP nick-end labeling (TUNEL), and transmission electronic microscope for ultrastructural changes in the cells (Gibbons et al., 2009). We previously have shown that apoptosis of ICC could be observed using whole-mount immunostaining for TUNEL in intramuscular, myenteric, and submucosal layers of stomach wall in diabetic rats (Chen et al., 2013a). In this study, we used a longitudinal section for c-kit immunolabeling to measure apoptotic ICC in the autumn and found that apoptotic ICC was increased in diabetic rats, while the number of apoptotic ICC was decreased with EA intervention. This implied that EA could reduce the apoptosis of ICC and maintain ICC function, resulting in an improved gastric motility.

IGF-1/IGF-1R signaling is an important pathway to regulate the survival and maintenance of ICC. Evidence suggests that progenitors of ICC fail to differentiate into mature cells without IGF-1 and/or insulin, and the maintenance of ICC phenotype and function are severely affected in the absence of IGF-1 and/or insulin (Horváth et al., 2006; Lorincz et al., 2008). It is also reported that the expression of IGF-1 was significantly decreased in patients with type 1 and type 2 diabetes (AboElAsrar et al., 2012). Similarly, loss of ICC was relevant to the deficiency of IGF-1 and/or insulin in diabetic murine (Horváth et al., 2005). Also, elevated IGF-1/IGF-1R signaling could inhibit the apoptosis of different cells, such as glioblastoma cells and renal carcinoma cells (Tracz et al., 2016; Zhang et al., 2018). It is consistent with our study that the IGF-1/IGF-1R signaling was distinctly reduced in the diabetic rats with lots of apoptotic ICC, while EA could improve the IGF-1/IGF-1R signaling and decrease the quantity of apoptotic ICC, indicating that EA partly activates the IGF-1/IGF-1R signaling to prevent the apoptosis of ICC.

Nrf2 is identified as a protective factor that adjusts the expression of proteins for the antioxidant and anti-inflammatory role (Ma, 2013). HO-1, an Nrf2-regulated gene, has important respects in antioxidant, anti-inflammatory, and anti-apoptotic in different cells (Araujo et al., 2012). Loboda et al. (2016) reported that the Nrf2/HO-1 pathway could prevent against oxidative stress-induced DNA damage and apoptosis. A previous study showed that HO-1 did not increase in the diabetic mice with gastroparesis, but induction of HO-1 could protect ICC and reverse diabetic gastroparesis (Choi et al., 2008; Mogami et al., 2013). Further study documented that EA at ST36 and BL13 could availably reduce lung injury through activation of the Nrf2/HO-1 pathway in a rabbit model (Yu et al., 2014). Similarly, Tian et al. (2018) suggested that EA could maintain the expression of HO-1 in diabetic gastroparesis mice. In our study, we found that the expression of the Nrf2/HO-1 pathway was not increased in diabetic rats, and furthermore, EA significantly improved the expression of the Nrf2/HO-1 pathway, meaning that EA may take effects on the Nrf2/HO-1 pathway to reduce ICC apoptosis in diabetic gastroparesis rats.

## Conclusion

The present study indicated that apoptotic ICC was found in the gastric antrum of diabetic rats, with decreased IGF-1/IGF-1R signaling and abnormal Nrf2/HO-1 pathway, resulting in disturbed contraction of the gastric antrum. Fortunately, EA with different frequencies could decrease the apoptosis of ICC, possibly through the activation of IGF-1/IGF-1R signaling, and improvement of the Nrf2/HO-1 pathway, and further improve the gastric contraction. Therefore, these results suggested that EA might be a potential therapeutic method for diabetic gastric motility dysfunction.

## Data Availability Statement

The original contributions presented in the study are included in the article/supplementary material, further inquiries can be directed to the corresponding authors.

## Ethics Statement

The animal study was reviewed and approved by the Animal Care and Use Committee of Binzhou Medical University Hospital Laboratory Animal Ethical Committee.

## Author Contributions

YC, HW, and KZ performed the experiments. YC wrote the manuscript. NS and QN gave suggestions on the discussion and interpretation of the data. CL was the guarantor of the whole lab and gave suggestions in the study and also took responsibility for the integrity of the data and the accuracy of the data analysis. All authors contributed to the article and approved the submitted version.

## Conflict of Interest

The authors declare that the research was conducted in the absence of any commercial or financial relationships that could be construed as a potential conflict of interest.
